# Supplementary material for: Integrated linkage-driven dexterous anthropomorphic robotic hand
Source: Nat Commun. 2021 Dec 14;12:7177. doi: 10.1038/s41467-021-27261-0 (PMC8671524; doi:10.1038/s41467-021-27261-0)
Supplement: Supplementary file 1 — Supplementary Information [file 41467_2021_27261_MOESM1_ESM.pdf]

SUPPLEMENTARY MATERIALS

Integrated Linkage-Driven Dexterous Anthropomorphic Robotic Hand

AUTHORS

Uikyum Kim[1,2,5]*, Dawoon Jung[3,5], Heeyoen Jeong[4], Jongwoo Park[2], Hyun-Mok Jung[2], Joono Cheong[3], Hyouk Ryeol Choi[4], Hyunmin Do[2], Chanhun Park[2]

AFFILIATIONS

[1] Department of Mechanical Engineering, Ajou University, Suwon, Korea 16499
[2] Department of Robotics & Mechatronics, Korea Institute of Machinery & Materials (KIMM), Daejeon, Korea 34103
[3] Department of Control and Instrument Engineering, Korea University, Sejong, Korea 30019
[4] Department of Mechanical Engineering, Sungkyunkwan University, Suwon, Korea 16419
[5] These authors contributed equally: Uikyum Kim, Dawoon Jung
*Corresponding author (uikykim@gmail.com)

This document contains the following sections:

- Supplementary Text 1. Definition of the features of the robotic hand
- Supplementary Text 2. Inverse kinematics of the two four-bar linkages
- Supplementary Text 3. Design of the bell crank in the mechanism
- Supplementary Text 4. Contact force-sensing strategy of the fingertip sensor
- Supplementary Text 5. Analysis of the experimental results during reliability tests
- Supplementary Text 6. Experimental data obtained during tool manipulation with scissors and tweezers
- Supplementary Text 7. Comparison of the robotic hand and human hand
- Supplementary Discussion
  Supplementary Fig. 1. Free body diagrams related to the bell-crank design. **a** PSU chain (PIP). **b** Four-bar linkage (PIP).
- Supplementary Fig. 2. Torque analysis at the PIP joint applied according to each joint angle by the bell-crank design. Cases for MCP angles of **a** 0°, **b** 22.5°, **c** 45°, **d** 67.5°, **e** 90°, and **f** 0–90°.
- Supplementary Fig. 3. Fabrication of the finger. **a** Mechanical parts. **b** Assembly process.
- Supplementary Fig. 4. Detailed dimensions of the hand
- Supplementary Fig. 5. Directional and locational relationship between the measured F/T and the contact force at the fingertip
- Supplementary Fig. 6. Grasping tests with YCB objects. **a** Adducted thumb. **b** Large diameter. **c** Power sphere. **d** Small diameter. **e** Tripod. **f** Three-finger pinch. **g** Precision disk. **h** Two-finger pinch.
- Supplementary Fig. 7. Measured contact force data while picking up, moving, and releasing objects with tweezers
- Supplementary Fig. 8. Definition of the length of the human hand

- Table S1. Specifications of a previously developed dexterous robotic hand with an ILDA hand
- Table S2. Comparison of the robotic hand and human hand

Other Supplementary Videos for this manuscript includes the following:

- Supplementary Video 1 (.mp4). Motions of the ILDA hand.
- Supplementary Video 2 (.mp4). Power grasping tests of the ILDA hand.
- Supplementary Video 3 (.mp4). Grasping various objects.
- Supplementary Video 4 (.mp4). Cutting a paper using scissors.
- Supplementary Video 5 (.mp4). Handling small objects using tweezers.

Supplementary Text 1. Definition of the features in the robotic hand.

Dexterity – Because the active motion of a human finger has 3-DOF [34], we define high dexterity as that with more than 3-DOF per finger. A human-level DOF is required to imitate movements mainly used by humans or to manipulate tools.

Fingertip force – The fingertip force of a human (based on the index finger) is defined as the high fingertip force. In previous studies[35], the human fingertip force has been reported as 27.9 N. As presented in Supplementary Table 1, it is very meaningful to achieve high fingertip forces and payloads because these are low for most of the dexterous robotic hands.

Controllability – Many types of robotic hands use compliant parts, such as springs, to efficiently transmit the driving force. It is difficult to completely model the characteristics of the mechanism using these compliant parts. There are a relatively large number of disturbance factors with a level of uncertainty in terms of control, compared to those in the case of mechanisms without compliant parts. Therefore, special control methods to solve the disturbance factors are required[36,37]. Among the currently developed robotic hands, there are many under-actuated robotic hands that use the adaptive grasping function. Because their structures may cause position errors owing to their compliance when force is applied in a specific situation (e.g., pinch grasp), special control methods are required, or the use is limited to a specific situation. Therefore, if all movements are implemented such that these are dependent on links, the occurrence of such errors is low, and the control is intuitive and simple.

Maintenance – The proposed mechanism provides easier maintenance than that of the tendon-driven mechanism. The hands based on the tendon-driven mechanism are rather difficult to assemble, and the problem of tendon-wire loosening often occurs with longer periods of use. Reassembly for its maintenance is not simple. Difficulties are encountered in the maintenance owing to an increase in cost. In contrast, the proposed mechanism is composed of a linkage structure, simplifying the assembly and maintenance.

Compactness – It describes the need to implement the shape of a hand as all parts (linkage structure, motors, sensors, and controller) are compactly integrated.

To check the performance of the developed hand based on the features described earlier, the performance indicators of the previously developed dexterous robotic hands are summarized in Supplementary Table 1.

Supplementary Text 2. Inverse kinematics of the two four-bar linkages.

The position vectors of A_i and B_i in the global coordinate frame, $O-xyz$ are denoted as $\mathbf{a}_i$ and $\mathbf{b}_i$, respectively. Vector $\mathbf{b}_i'$ is $\mathbf{b}_i$ expressed in the moving coordinate frame, $P-uvw$. Vector $\mathbf{d}_i(=[0 \quad 0 \quad d_i]^T$ for $i=1,2,3)$ is the position vector of the prismatic joints, and vector $\mathbf{p}(=[0 \quad 0 \quad p]^T)$ is the position vector related to the origin of $P-uvw$. Here, d_i is the displacement input of the ith prismatic joint. Vector $\mathbf{l}_i$ is the position vector from point B_i to the spherical joint of the ith PSS chain. Because points A_i and B_i are symmetrical to the x- and v-axes, respectively, $\mathbf{a}_1=[a_x \quad a_y \quad 0]^T$, $\mathbf{a}_2=[-a_x \quad a_y \quad 0]^T$, $\mathbf{b}_1'=[b_x \quad b_y \quad 0]^T$, and $\mathbf{b}_2'=[-b_x \quad b_y \quad 0]^T$. For analyzing the inverse kinematics of the PSS chains, the vector loop equation was derived from the geometry of each PSS chain as follows:

$$\mathbf{a}_i+\mathbf{d}_i=\mathbf{p}+\mathbf{R}\mathbf{b}_i'+\mathbf{l}_i \quad (\text{for } i=1,2). \tag{S1}$$

Since $\mathbf{l}_i{}^T\mathbf{l}_i=l_i{}^2$, from Eq. 2, the following equation can be obtained:

$$l_i{}^2=\left(\mathbf{a}_i+\mathbf{d}_i-\mathbf{p}-\mathbf{R}\mathbf{b}_i'\right)^T\left(\mathbf{a}_i+\mathbf{d}_i-\mathbf{p}-\mathbf{R}\mathbf{b}_i'\right). \tag{S2}$$

Because $l_i{}^2$ is a constant related to the length of the link, it can be calculated. When $\mathbf{k}_i=\mathbf{p}+\mathbf{R}\mathbf{b}_i'-\mathbf{a}_i$ is considered, all the components of $\mathbf{k}_i(=[k_{i,x} \quad k_{i,y} \quad k_{i,z}]^T)$ are known. Therefore, from Eq. S2, which is a quadratic equation for the displacements of prismatic joints, d_i can be summarized as Eq. 2 in the main text.

The mechanism related to the motions of the PIP and DIP joints consists of one PSU chain and two crossed four-bar linkages (Fig. 3). In the first step, the inverse kinematics of the PSU chain were analyzed (Fig. 3b). $P_0(=[0 \quad c_y \quad 0]^T)$ represents the intersection between the center of the third prismatic joint and the xy plane. P_3 represents the revolute joint attached to the moving platform. P_1 and P_2 represent the spherical and universal joints, respectively. Position vector $\mathbf{P}_{01}(=[0 \quad 0 \quad d_3]^T)$ can be calculated as P_1-P_0. With the rotational matrix $(\mathbf{R})$, position vectors $\mathbf{P}_{23}$ and $\mathbf{P}_{03}$ in the global coordinate frame, $O-xyz$, are expressed as

$$\mathbf{P}_{23}=\mathbf{R}\mathbf{P}_{23}'=\begin{bmatrix} l_4Sq_2S(q_1-\alpha) \\ l_4C(q_1-\alpha) \\ l_4Cq_2S(q_1-\alpha) \end{bmatrix}, \mathbf{P}_{03}=\mathbf{R}\mathbf{P}_3'-P_0=\begin{bmatrix} hSq_2Sq_1 \\ hCq_1-c_y \\ hCq_2Sq_1+p \end{bmatrix}. \tag{S3}$$

Here, $\mathbf{P}_{23}'$ and P_3' are the position vector and the point expressed in the local frame, $P-uvw$, respectively, h is the length from the origin of the local coordinate frame to point P_3', l_4 is the length of link P_{23}, and α is the angle between the bell-crank and the moving platform. With these position vectors, the following vector loop equations were derived:

$$\mathbf{P}_{01}+\mathbf{P}_{12}+\mathbf{P}_{23}=\mathbf{P}_{03}. \tag{S4}$$

From Eq. S4, position vector $\mathbf{P}_{12}$ was derived as

$$\mathbf{P}_{12} = \begin{bmatrix} hSq_2Sq_1 - l_4Sq_2S(q_1 - \alpha) \\ hCq_1 - l_4C(q_1 - \alpha) - c_y \\ hCq_2Sq_1 - l_4Cq_2S(q_1 - \alpha) + p - d_3 \end{bmatrix} = \begin{bmatrix} P_{12,x} \\ P_{12,y} \\ P_{12,z} - d_3 \end{bmatrix}. \quad \text{(S5)}$$

As $||\mathbf{P_{12}}|| = l_3$ and $\mathbf{l}_3{}^T\mathbf{l}_3 = l_3{}^2$, the following equation can be obtained:

$$P_{12,x}{}^2 + P_{12,y}{}^2 + (P_{12,z} - d_3)^2 = l_3{}^2. \quad \text{(S6)}$$

Eq. S6 can be summarized in the descending order for d_3. Thus, the result is as Eq. 3 in the main text.

At Eq. 3, $A_1 = P_{12,z}$ and $B_1 = P_{12,x}{}^2 + P_{12,y}{}^2 + P_{12,z}{}^2 - l_3^2$. Here, angle α is a variable related to the flexion and extension of the PIP joint (Fig. 3c). Therefore, based on the motion of the PIP joint, the displacement input, d_3, was determined.

In Fig. 3c, because $\beta_1(= 2\pi/9)$ is constant, angle α and that of the four-bar linkage (θ_1) have the following relationship: $\alpha = 5\pi/6 - \theta_1$. Therefore, if the relationship between the angle of the PIP joint (θ_3) and θ_1 is analyzed, the relationship with d_3 is also obtained. The position vector of the links is denoted as $\boldsymbol{r}_i$, and the angles between these links and the v-axis of the $v - w$ coordinate system are denoted as θ_i. With this four-bar linkage, the vector loop equation is represented as $\mathbf{r}_1 + \mathbf{r}_2 + \mathbf{r}_3 = \mathbf{r}_4$. The two equations for the horizontal and vertical components of this equation as follows:

$$\begin{cases} r_1C\theta_1 + r_2C\theta_2 + r_3C\theta_3 = r_4C\theta_4 \\ r_1S\theta_1 + r_2S\theta_2 + r_3S\theta_3 = r_4S\theta_4 \end{cases}. \quad \text{(S7)}$$

In Eq. S7, eliminating θ_2 gives

$$A_2S\theta_1 + B_2C\theta_1 = C_2. \quad \text{(S8)}$$

Eq. S8 can be solved for θ_1 as follows:

$$\theta_1 = \sin^{-1}\frac{C_2}{\sqrt{A_2{}^2 + B_2{}^2}} - \tan^{-1}\frac{B_2}{A_2}, \quad \text{(S9)}$$

where $A_2 = 2r_1(r_3S\theta_3 - r_4S\theta_4)$, $B_2 = 2r_1(r_3C\theta_3 - r_4C\theta_4)$, and $C_2 = r_2{}^2 - r_1{}^2 - r_3{}^2 - r_4{}^2 + 2r_3r_4C\theta_{3-4}$.

Because joints P_3' and P_6' are fixed, θ_4 is constant. Therefore, using Eq. 3 and Eq. S9, the relationship between the angle of the PIP joint and the displacement input of the prismatic joint was investigated. To maintain consistency with the previous notation (q_1, q_2), θ_3 (the angle of the PIP joint) is denoted as q_3. Next, the motion of the DIP joint that is dependent on the motion of the PIP joint was analyzed. Between the PIP and DIP joints, there is a four-bar linkage, as shown in Fig. 3d. In the figure, P_i' denotes P_i that is expressed in coordinate frame $P - uvw$. Point P_6' represents the PIP joint, and point P_9' represents the DIP joint. Joints P_6' and P_7' are

fixed. The position vectors of the links are denoted as $\mathbf{u}_i$, and the angles of the vectors are denoted as γ_i. Then, the vector loop equation of the above four-bar linkage is $\mathbf{u}_1 + \mathbf{u}_2 = \mathbf{u}_3 + \mathbf{u}_4$. Using the method described earlier, the following two equations were derived for the vector loop:

$$\begin{cases} u_1 C\gamma_1 + u_2 C\gamma_2 = -u_3 + u_4 C\gamma_4 \\ u_1 S\gamma_1 + u_2 S\gamma_2 = u_4 S\gamma_4 \end{cases}. \quad \text{(S10)}$$

In addition, the equation where γ_4 is eliminated and θ_2 is considered is as follows:

$$A_3 S\gamma_2 + B_3 C\gamma_2 = C_3, \quad \text{(S11)}$$

where

$$\begin{gathered} A_3 = 2u_1u_2S\gamma_1, \\ B_3 = 2u_1u_2C\gamma_1 + 2u_2u_3, \\ C_3 = u_4^2 - u_1^2 - u_2^2 - u_3^2 - 2u_1u_3C\gamma_1. \end{gathered} \quad \text{(S12)}$$

Then, the following is obtained from Eq. S11:

$$\gamma_2 = sin^{-1}\frac{C_3}{\sqrt{A_3^2+B_3^2}} - tan^{-1}\frac{B_3}{A_3}. \quad \text{(S13)}$$

Using the result, the relationship between γ_1 and γ_2 can be analyzed. Next, angle γ_1 is also related to θ_3, the PIP joint angle, as shown in Fig. 3d.

According this figure, equation $\gamma_1 = \theta_3 - \beta_2 + \beta_3$ can be derived using the geometrical relationships of the finger. In this equation, β_2 and β_3 are constant because link P_{67}' and triangle frame P_{567}' are constant in the $v - w$ coordinate frame. Equation S7 shows that angle γ_2, the DIP joint angle, is dependent on q_3, the PIP joint angle. Consequently, the inverse kinematic relations between the angles of the three joints and the three linear displacements can be expressed in a closed-form equation.

Supplementary Text 3. Design of the bell-crank in the mechanism.

A bell crank is responsible for changing motion through its angle in a limited space. According to the characteristics of the linkage-driven mechanism, the length of a moment arm is changed by the joint angle. Therefore, the magnitude of the torque generated when a constant force in one direction is applied to the end of the moment arm varies according to the position of the joint. When the link structure becomes more complex, such as a structure connected from d_3 to the PIP joint in Fig. 3b and c, the torque distortion becomes more severe. Therefore, we used the bell-crank mechanism to compensate for the torque distortion and obtain the largest torque in the desired position of the PIP joint.

To calculate the transmission efficiency using the included angle of the bell crank as a variable, we analyzed the free body diagram of the linkage chains from d_3 to the PIP joint, as presented in Supplementary Fig. 1.

The kinematic chains of the PIP joint from d_3 could be divided into two components based on fixed position P_3, as shown as Supplementary Fig. 1. At this time, the equivalent forces of one linkage chain (Supplementary Fig. 1a) are

$$\begin{cases} -F_{d_3} + F_{1,z} = 0 \\ l_{3,y}F_{1,z} - l_{3,z}F_{1,y} = 0 \\ \tau_\alpha + l_{4,y}F_{1,z} - l_{4,z}F_{1,y} = 0 \end{cases}, \tag{S14}$$

where $\mathbf{l}_3, \mathbf{l}_4$ are the vectors of each link length in first linkage chain, τ_α is the torque of the bell crank onto P_3. The magnitudes of applied force F_1 are equal for the links, and the direction of F_1 is along that of the open-loop link. From Eq. S14, torque τ_α is calculated as

$$\tau_\alpha = \left(\frac{l_{4,z}l_{3,y} - l_{4,y}l_{3,z}}{l_{3,z}}\right) \cdot F_{d_3}. \tag{S15}$$

Next, the equivalent forces of the other linkage chain (Supplementary Fig. 1b) are

$$\begin{cases} \tau_\alpha + r_{1,y}F_{2,z} - r_{1,z}F_{2,y} = 0 \\ r_{2,y}F_{2,z} - r_{2,z}F_{2,y} = 0 \\ -\tau_{pip} + r_{3,y}F_{2,z} - r_{3,z}F_{2,y} = 0 \end{cases}, \tag{S16}$$

where $\mathbf{r}_1, \mathbf{r}_2$ are the vectors of each link length in the linkage chain. Further, the magnitudes of applied force F_2 are equal for the links, and the direction of F_2 is along that of the open-loop link. From Eq. S16, torque τ_{pip} is calculated as

$$\tau_{pip} = \left(\frac{r_{2,z}r_{3,y} - r_{2,y}r_{3,z}}{r_{1,z}r_{2,y} - r_{1,y}r_{2,z}}\right) \cdot \tau_\alpha. \tag{S17}$$

The torque transmission rate varies according to the joint position owing to the design of the bell crank. Specifically, among the various design parameters of the bell crank, adjusting the included angle (β_1) of the bell crank in terms of its design is easy.

Therefore, we analyzed the efficient torque transmission structure using the bell crank as the design parameter.

The precise point at which the joint of a human finger can generate the strongest gripping force is not known. However, previous studies have shown that the gripping force is stronger in the bent pose than in the stretched pose[44]. Therefore, when each joint angle is in an intermediate position (MCP: 45°, PIP: 45°), securing the strongest gripping force is effective. When manipulating or gripping various objects, the most used joint angle is the intermediate position[45]. Hand synergies constituting the gripping action are also mainly distributed around the intermediate position[46]. Therefore, we analyzed the bell-crank design to secure the highest torque in the intermediate position.

To calculate the transmission efficiency using the included angle of the bell crank as a variable, we analyzed the free-body diagram of the linkage chains from d_3 to the PIP joint, as presented in Supplementary Fig. 1. A detailed equation derivation for this has been presented in Supplementary Text 3. As a result, a relation of torque (τ_{pip}) generated by the input force (F_{d_3}) to d_3 was derived. As presented in Supplementary Fig. 2a-e, the angle of the MCP joint was analyzed by dividing it into five cases. The graphs show the torque values (τ_{pip}) from 0° to 90° of the PIP joint when an input (F_{d_3}) of 1 N is applied. The torque values are analyzed for three cases of the included angle: 0° (without the bell-crank part), 40°, and 80°. Supplementary Fig. 2a presents the torque data of the MCP joint in the full extension pose, and Supplementary Fig. 2e presents the torque data of the MCP joint in the full flexion pose. When the angle of the MCP and PIP joints was 45°, the included angle of the bell crank that secures the highest torque was confirmed to be 40°, as displayed in Supplementary Fig. 2c. In addition, Supplementary Fig. 2f represents the sum of torques generated in all MCP joint positions (0° ~ 90°) according to the PIP angle. The total torque can be derived from $\int_0^{\pi/2} \tau_{pip} q_2 dq_2$. In the graph, when the ball-crank angle is 40°, the highest total torque is secured at a PIP angle of 45°. Further, it is possible to obtain the torque with the smallest fluctuation in the entire section of the PIP joint.

As a result, we found the optimal value of the included angle of the bell crank that can provide the highest gripping force in the 45° bent pose while maintaining high gripping forces in the full extension (stretched) and full flexion poses of all the joints.

Supplementary Text 4. Contact force-sensing strategy of the fingertip sensor.

Using the wrench measurements obtained from the force sensor connected to the fingertip, we could estimate the position of the contact point.

A method to estimate the contact point using force measurements was proposed by Bicchi et al.[54]. Let us assume that the contact point is a contact model with friction. When a contact force $\mathbf{P}$ is applied to a contact position, the measured six-axis F/T by the fingertip sensor is represented as (Supplementary Fig. 5)

$$\begin{bmatrix} \mathbf{f} \\ \mathbf{t} \end{bmatrix} = \begin{bmatrix} \mathbf{R}_{\mathrm{c}}^{\mathrm{s}} \\ S(\mathbf{r}_{\mathrm{c}})\mathbf{R}_{\mathrm{c}}^{\mathrm{s}} \end{bmatrix} \mathbf{P}, \tag{S18}$$

where $\mathbf{R}_{\mathrm{c}}^{\mathrm{s}}$ is the rotation matrix obtained from the contact and sensor frames, r_c is the distance vector between the contact point and the position of the force sensor, and $S(\mathbf{A})$ is the skew-symmetric matrix of vector $\mathbf{A}$. We can rearrange equation $\mathbf{r}_{\mathrm{c}}$ as

$$\mathbf{r}_{\mathrm{c}} = (S(\mathbf{f}))^{\dagger}\mathbf{t}, \tag{S19}$$

where $\mathbf{A}^{\dagger}$ is the pseudo-inverse of matrix $\mathbf{A}$. The skew-symmetric matrix is rank deficient, and the pseudo-inverse of the skew-symmetric matrix should have one homogenous solution. Therefore, distance $\mathbf{r}_{\mathrm{c}}$ can be obtained as follows:

$$\mathbf{r}_{\mathrm{c}} = \mathbf{r}_{\mathrm{c,p}} + \lambda \mathbf{r}_{\mathrm{c,h}}, \tag{S20}$$

where

$$\mathbf{r}_{\mathrm{c,p}} = (\mathbf{f}/||\mathbf{f}||) \times \mathbf{t}, \mathbf{r}_{\mathrm{c,h}} = \mathbf{f}/||\mathbf{f}||, \tag{S21}$$

where λ is the parameter of the homogenous solution. The range of $\mathbf{r}_{\mathrm{c}}$ represents a line parameterized by λ. This line intersects the convex surface of the fingertip at the contact point.

We estimated the position of the contact point by obtaining the solution of the line and surface equations. We designed the surface of the fingertip as a combination of general forms of surfaces. The general forms of surfaces that we used were a sphere, cylinder, and plane. Each type of surface is defined by minimal parameters. The surface equation described in the surface frame is

$$\mathrm{Surface}_i(\mathbf{r}_{\mathrm{sf}_i}) = \mathbf{r}_{\mathrm{sf}_i}^{\mathrm{T}} \mathbf{A}^T \mathbf{A} \mathbf{r}_{\mathrm{sf}_i} - R^2 = 0, \tag{S22}$$

where $\mathbf{r}_{\mathrm{sf}_i} = \mathbf{T}_{\mathrm{s}}^{\mathrm{sf}} \mathbf{r}_{\mathrm{c,p}} + \lambda \mathbf{T}_{\mathrm{s}}^{\mathrm{sf}} \mathbf{r}_{\mathrm{c,h}} = \mathbf{r}_{\mathrm{sf}_i,\mathrm{p}} + \lambda \mathbf{r}_{\mathrm{sf}_i,\mathrm{h}}$,

$$\mathbf{A} = \begin{bmatrix} 1/R_x & 0 & 0 \\ 0 & 1/R_y & 0 \\ 0 & 0 & 1/R_z \end{bmatrix}, \tag{S23}$$

where $\mathbf{T}_{\mathrm{s}}^{\mathrm{sf}}$ is the transformation matrix between the sensor and surface frames. The general solution of the ith surface equation is

$$\lambda_i = \frac{-\mathbf{r}_{\mathrm{sf}_i,\mathrm{h}}^T\mathbf{A}_\mathrm{p} \pm \sqrt{(\mathbf{r}_{\mathrm{sf}_i,\mathrm{h}}^T\mathbf{A}_\mathrm{p})^2 - \mathbf{r}_{\mathrm{sf}_i,\mathrm{h}}^T\mathbf{A}_\mathrm{h}(\mathbf{r}_{\mathrm{sf}_i,\mathrm{p}}^T\mathbf{A}_\mathrm{p} - R^2)}}{\mathbf{r}_{\mathrm{sf}_i,\mathrm{h}}^T\mathbf{A}_\mathrm{h}}, \tag{S24}$$

where $\mathbf{A}_\mathrm{P} = \mathbf{A}^T\mathbf{A}\boldsymbol{r}_{\mathrm{sf}_i,\mathrm{p}}$ and $\mathbf{A}_\mathrm{h} = \mathbf{A}^T\mathbf{A}\mathbf{r}_{\mathrm{sf}_i,\mathrm{h}}$. Thus, the position of the contact point in the surface frame is

$$\mathbf{r}_{\mathrm{sf}_i}' = \mathbf{T}_{\mathrm{sf}}^{\mathrm{s}}\mathbf{r}_{\mathrm{sf}_i,\mathrm{p}} + \lambda_i\mathbf{T}_{\mathrm{sf}}^{\mathrm{s}}\mathbf{r}_{\mathrm{sf}_i,\mathrm{h}}. \tag{S25}$$

The estimated contact point is determined when the given surface has a convex shape. However, the fingertip has many surfaces, and each surface is formed in a given range. Therefore, we analyzed two conditions to confirm if the true contact point was identified. Each point on the surface could be described in spherical, cylindrical, or Cartesian coordinates, and we could calculate the coordinate values of the contact point.

Supplementary Text 5. Analysis of the experimental results during reliability tests.

The experimental results were analyzed by performing several experiments related to the reliability of the hand as follows:

(1) **Long-time operation of the hand**. We tested whether the hand can stably perform repeated movements for a prolonged period. We repeated free motions of five fingers of the hand for 30 min. It was confirmed that normal operation was performed during the experiment. The long-time operation test is illustrated in Supplementary Video 1.

(2) **Constant fingertip force applied by the finger**. To test whether a fingertip force can be continuously applied by the developed robotic finger, an experiment was performed, wherein a fingertip force was applied by a finger for 30 min in the experimental environment displayed in Fig. 6a. Fig. 7a shows the measured fingertip force and the temperature of the hand. For accurate measurement, the force was measured by the reference sensor, and the part with the highest temperature on the motor control board was measured every 30 s using a temperature-measuring device (TESTO845, TESTO). The finger was allowed to press the sensor for 30 min based on position control with static-force analysis. Experimental results were obtained; the fingertip force measured by the reference sensor continuously decreased from 13.5 N to 11.2 N. One of the reasons for this decrease is thought to be the relaxation effect due to the soft material of the fingertip. For a detailed analysis, a repeatability test was performed. The temperature was measured to be 38.5 °C in the no-load state; it continued to increase over time and converged to 62 °C in 28 min. The current measured during the experiment was constant (0.3 A).

(3) **Grasping a ball for a prolonged period**. An experiment was conducted to measure the heat and current generated in the entire robotic hand. A soft ball with a diameter of 120 mm was held for 10 min while applying a fingertip force of 20 N with each of the five fingers. Fig. 7b and Supplementary Video 2 show the measured temperature and current during the experiment. The current driving the robotic hand was almost constant (0.9 A). The measured temperature gradually increased and converged to approximately 65 °C. It operated stably even though all the fingers were holding the ball with high force. The temperature increased more rapidly than when driving with one finger, and the measured temperature was higher, but the difference was not large.

(4) **Repeatability test**: Repeatability tests were performed on the robotic finger by repeating the full stretched and bent finger poses in the experimental environment displayed in Fig. 6a. Fig. 7c presents the force data measured during an experiment in which the force applied to the sensor for 5 s was repeated 60 times in 10 min. When the peak value of the force at each contact was defined as the contact force, the average contact force was 16.7 N, maximum error 0.4 N, and standard deviation 0.12 N in 60 contacts. Fig. 7d presents the force data measured during an experiment in which an applied force for 1 s was repeated 300 times in 11 minutes. The average of the measured contact forces was 14.62 N, maximum error 0.66 N, and standard deviation 0.18 N. As a result, it was possible to apply a constant force without abnormal motion of the hand in numerous repeated motions. The repeatability of movement of the developed robotic finger was investigated by deriving the location of contact between the finger and sensor.

During these two experiments, fingertip contact location data were derived. As shown in Fig. 7e and f, the contact locations were derived from the measured 6-axis F/T data using the reference sensor. As a result, the standard deviation of the distance error of 60 contact points was 0.14 mm, and the standard deviation of the error of 300 contact points was 0.099 mm. The developed robot exhibited superior repetition performance in both experiments.

(5) **Payload test**. We conducted a payload test with dumbbells of various weights. Intuitively, it was expected that the hand would deliver superior payload performance because it is sturdy and offers a large fingertip force. Supplementary Video 2 and Fig. 7g and h indicate that the developed hand grasps and lifts the dumbbells (7, 12, and 18 kg). It was possible to hold an 18-kg dumbbell while lifting it up and down. Because the highest payload among the investigated robotic hands was 9 kg, superior payload performance of the developed hand was demonstrated.

Supplementary Text 6. Experimental data obtained during tool manipulation with scissors and tweezers.

The force data measured when handling an object with tweezers are presented in Supplementary Fig. 7. As seen in Supplementary Video 5, the thumb and index finger are mainly used to manipulate the tweezers, and the data measured by the fingertip sensors on the thumb and index finger are developed. The data are related to the grasping forces. It was confirmed that the measured forces increase when the robotic hand approaches and holds the tweezers. The force fluctuates when the tweezers are pulled from the cradle, and this fluctuation when changing the direction of the tweezers by the ringer finger was measured. Then, when the grasping force to open the tweezers was released, it reduced; the grasping force was confirmed to increase when releasing the finger mounted on the tweezers. By changing the position of the robot arm, we could measure the force during the grasping and peeling of the chip's cover film with tweezers. Lastly, holding the small chip with tweezers, the fluctuation in grasping force when moving the chip to the adjacent board was checked.

Supplementary Text 7. Comparison of the robotic hand and human hand.

The phalange lengths of an index finger, working range of each joint, palm size, and workspace volume are summarized in Supplementary Table 2. The definition of length of a human hand is presented in Supplementary Fig. 8. A survey was conducted based on the average male hand[48,49]. The length of the finger and the length and width of the palm of the robot were confirmed to be 5.56%, 16.79%, and 19.24% larger than those of a human, respectively. The maximum length of the robotic hand was found to be 12.93% larger than that of a human hand.

Supplementary Discussion.

Because of the nature of the linkage-driven mechanism, it is difficult to move each joint independently using a serial manipulator with continuous joints. In a tendon-driven mechanism, independent movement of each joint can be realized by precisely passing through the central axis of the joint where the tendons pass and connect to the next joint. If the thickness of the link is identical to that of the tendons, then the stiffness of this configuration is very low. Therefore, this paper proposed a mechanism configuration combining the 2-DOF parallel mechanism and the 1-DOF serial mechanism for enabling independent operation of each joint.

The linkage-driven mechanism is easy to construct in a structure with high stiffness, but the multi-DOF joints required to achieve 2-DOF in the MCP joint of the robotic hand tend to have low stiffness. Therefore, the stress distribution is effective when using rod ends as multi-DOF joints. In addition, the mechanism was designed to enable efficient power transmission through a power transmission part capable of minimizing friction. Therefore, we could ensure a high fingertip force and its

backdrivability. To the best of our knowledge, this type of hand has not been developed in previous studies.

When evaluated with five six-axis F/T sensors mounted on each fingertip of the hand to obtain tactile sensing capability, the wirings of the sensors were connected to the main MCU board through an empty space between the links. The measured force torque was converted into a triaxial contact force and the position of the force on the surface of the fingertip through the contact-force-sensing algorithm. Thus, we could measure the force during grasping or manipulation of an actual object.

All the five fingers of the hand had the same configuration to ensure ease of fabrication. In particular, if the electronic parts are extremely large or the wiring is not performed compactly, the advantages of the developed hand are lost. Thus, the board configuration and the wiring are compact. The wiring was effectively performed with a four-pin connector on the back of the hand.

Most delicate tasks can be covered with fingertip sensing alone. However, tactile sensing in intermediate areas is important for a power grasp. In the future, we will develop artificial skin technology capable of tactile sensing and conduct research to combine it with the intermediate areas such as palm of the robotic hand.

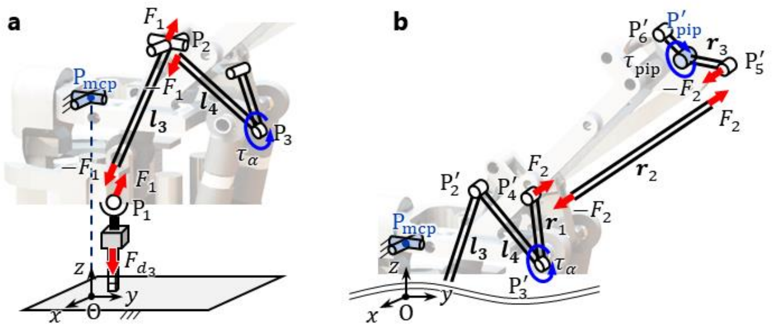

Supplementary Fig. 1. Free body diagrams related to the bell-crank design. **a** PSU chain (PIP). **b** Four-bar linkage (PIP).

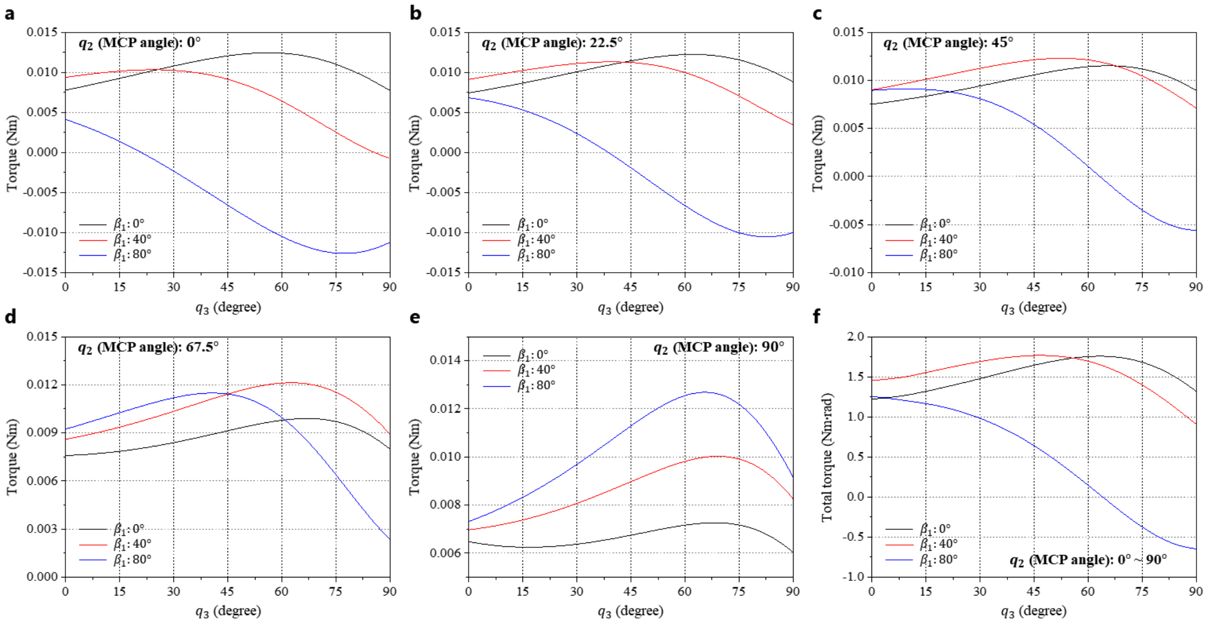

Supplementary Fig. 2. Torque analysis at the PIP joint applied according to each joint angle by the bell-crank design. Cases for MCP angles of **a** 0°, **b** 22.5°, **c** 45°, **d** 67.5°, **e** 90°, and **f** 0–90°.

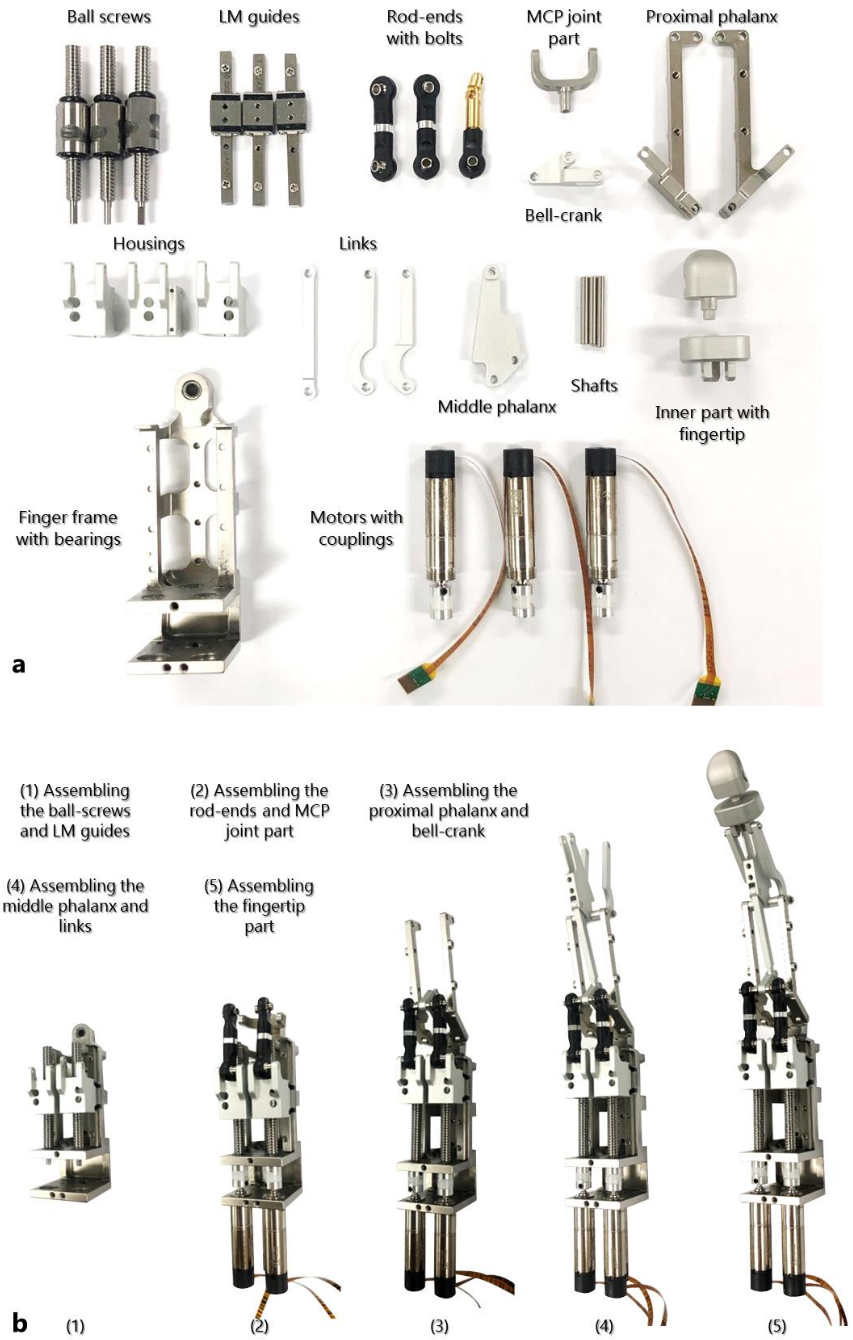

Supplementary Fig. 3. Fabrication of the finger. **a** Mechanical parts. **b** Assembly process.

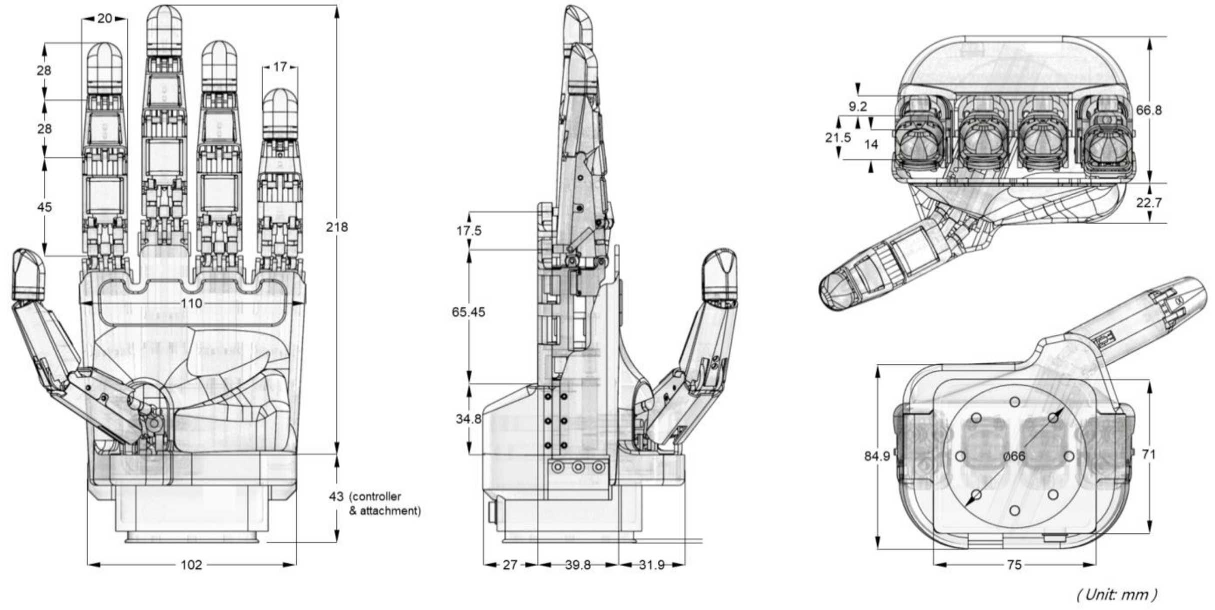

Supplementary Fig. 4. Detailed dimensions of the hand

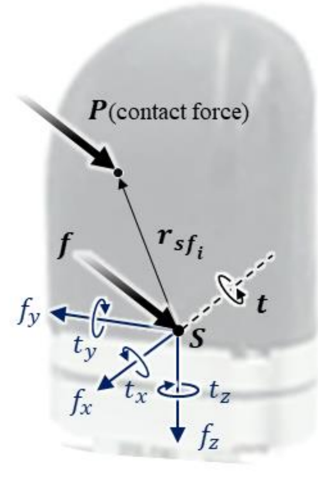

Supplementary Fig. 5. Directional and locational relationship between the measured F/T and the contact force at the fingertip

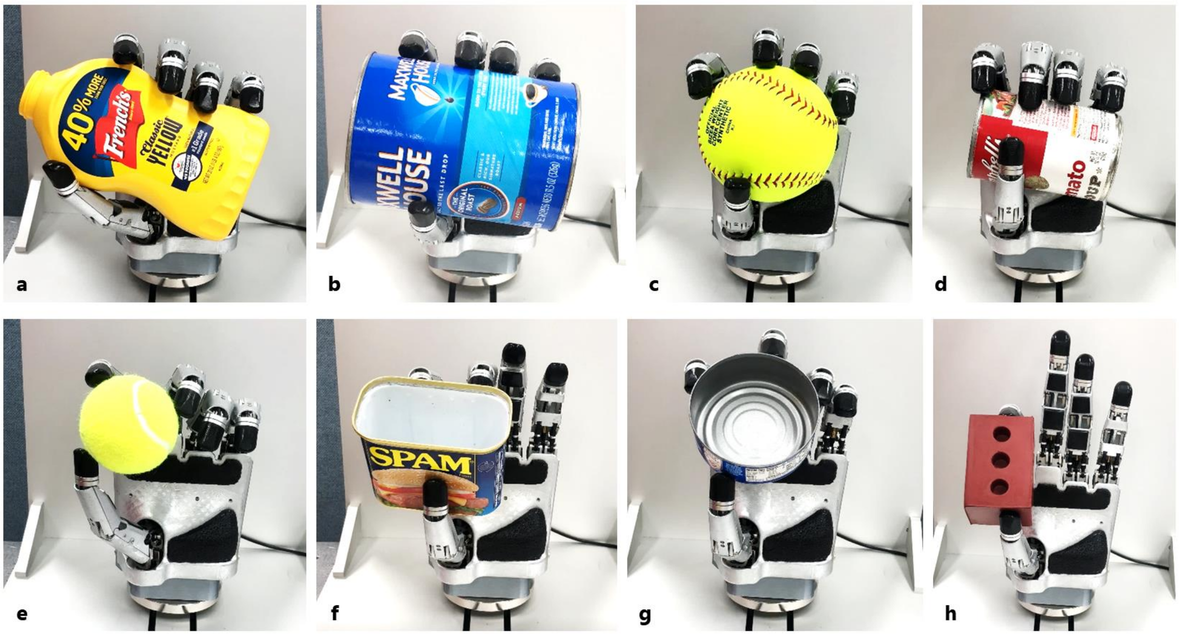

Supplementary Fig. 6. Grasping tests with YCB objects. **a** Adducted thumb. **b** Large diameter. **c** Power sphere. **d** Small diameter. **e** Tripod. **f** Three-finger pinch. **g** Precision disk. **h** Two-finger pinch.

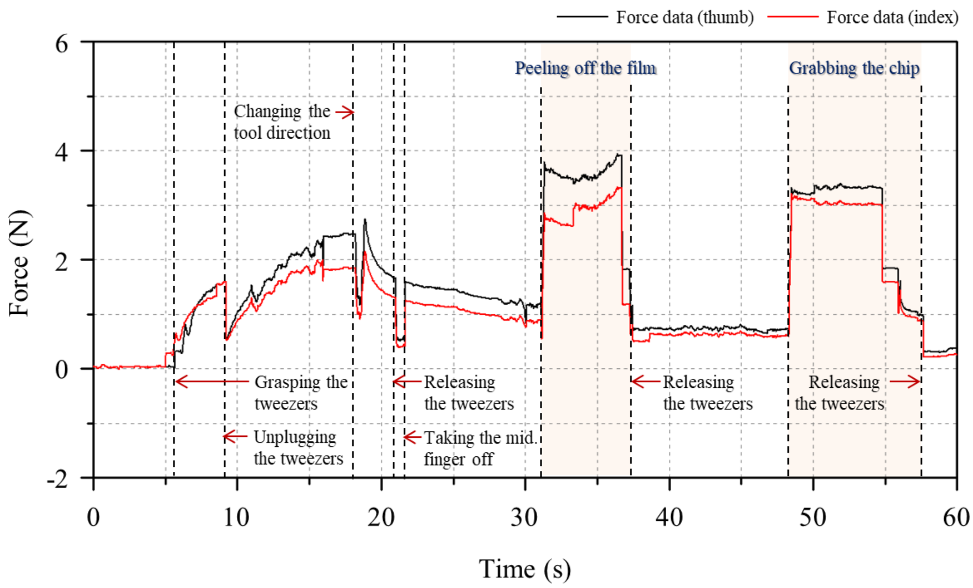

Supplementary Fig. 7. Measured contact force data while picking up, moving, and releasing objects with tweezers

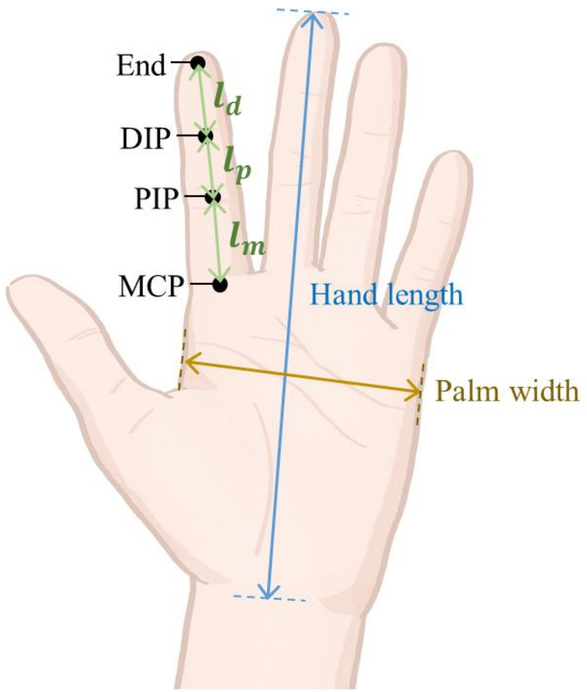

Supplementary Fig. 8. Definition of the length of the human hand

Supplementary Table 1. Specifications of previously developed dexterous robotic hand with ILDA hand.

	Active DOF / # of fingers	Active DOF of index finger	Finger length	Hand size	Forearm	Weight	Fingertip force	Payload	Driving type
			(mm)	(mm)		(kg)	(N)	(kg)	
ILDA hand	15/5	3	101	218	X	1.1	34	18	LD
DLR/HIT 2[13]	15/5	3	169.1	-	X	1.5	10	-	DM
David's hand[17]	20/5	4	-	-	O	4.5	40	-	TD
SVH[28]	9/5	2	112	243	X	1.3	-	-	LD
Shadow dexterous hand[18]	20/5	4	105	448	O	4.3	-	4	TD
CEA hand[38]	20/5	4	-	-	O	4.2	4.2	-	TD
DEXMART hand[39]	16/5	3	216	-	O	-	10	-	TD
Integrated robotic hand[40]	12/4	3	-	-	X	-	10	-	TD
RoboRay hand[41]	12/5	3	80	160	O	1.59	15	-	TD
Robonaut 2 Hand[16]	14/4	3	127	304	O	-	21.85	9	TD
NAIST hand[42]	12/5	3	150	-	X	-	5	-	TD
Allegro hand[43]	16/4	4	144	239	X	1.09	4	5	DM
Driving type: DM (Direct motored), TD (tendon driven), LD (linkage driven)									

Supplementary Table 2. Comparison of the robotic hand and human hand

	Finger length (index)				Finger joint range				Palm size		Max. hand length	Workspace volume
	l_p	l_m	l_d	Sum	MCP (abd/add)	MCP (flex/ext)	PIP	DIP	Length	Width		
	(mm)	(mm)	(mm)	(mm)	(deg)	(deg)	(deg)	(deg)	(mm)	(mm)	(mm)	(mm^3)
Human	48.3	28.2	19.1	95.6	±30	90	90	80	85.84	88.9	193.04	96149 (119800*)
Robot	45	28	28	101	±35	90	90	80	100.25	106	218	188740
The ratio between human and robotic hand												
Ratio	6.8%	0.7%	46%	5.56%					16.79%	19.24%	12.93%	96.30%

l_p: proximal phalange length, l_m: middle phalange length, l_d: distal phalange length.
MCP(abd/add): the abduction/adduction displacement angle of MCP joint
MCP(flex/ext): the flexion/extension displacement angle of MCP joint
*The calculated volume under same lengths with robotic finger
