# Supplementary material for: Integrated linkage-driven dexterous anthropomorphic robotic hand
Source: Nat Commun. 2021 Dec 14;12:7177. doi: 10.1038/s41467-021-27261-0 (PMC8671524; doi:10.1038/s41467-021-27261-0)
Supplement: Supplementary file 3 — Description of Additional Supplementary Files [file 41467_2021_27261_MOESM3_ESM.pdf]

Title: Supplementary Video 1
Description: Motions of the ILDA hand.

Title: Supplementary Video 2
Description: Power grasping tests of the ILDA hand.

Title: Supplementary Video 3
Description: Grasping various objects.

Title: Supplementary Video 4
Description: Cutting a paper using scissors.

Title: Supplementary Video 5
Description: Handling small objects using tweezers
